# Does formal workplace based assessment add value to informal feedback?

**DOI:** 10.15694/mep.2017.000027

**Published:** 2017-02-09

**Authors:** Janet Lefroy, Ashley Hawarden, Simon Gay, Robert McKinley

**Affiliations:** 1 Keele University School of Medicine

**Keywords:** Undergraduate medical education, Workplace based assessment, Feedback

## Abstract

This article was migrated. The article was marked as recommended.

Feedback is a key component of learning but effective feedback is a complex process with many aspects. One aspect may be a written summary which is passed to the learner but this may not be valued by learners. We examined the role of written feedback in the feedback process to determine whether it does more than provide a simple summary of the interaction. We conducted a secondary analysis of data gathered for a study of formative workplace based assessment. Interview data from 24 interviews with students and written summaries of workplace based assessments for 23 of them were reanalysed by two researchers who were already immersed in the data and examined all references to verbal, informal feedback and written, formal feedback or the assessment tool used. We found that students valued the verbal feedback discussion highly and that they often considered the written summaries superfluous. We also found that the act of preparing written feedback augmented the feedback discussion and tutors had adopted the language of the formal instrument in the verbal feedback and free text written feedback.

What this study adds to existing research is evidence that there may be a secondary faculty development effect of requiring the preparation of written feedback which has served to enhance the educational content of feedback. Although this is not proof of causality (the requirement to provide written feedback alone producing the positive effects), we consider that the likelihood is sufficiently strong to continue the practice.

## Background

Feedback to students in the clinical setting comes in a variety of forms including conversations with a clinical tutor following a student-patient encounter or case presentation; written feedback using instruments such as mini-CEX; formal clinical supervisor discussions which may involve a 360 degree appraisal; self- and peer-assessment. Many of these feedback opportunities are embedded within educational systems in which workplace-based assessment (WBA) is a required component and the formative potential can become lost in the ticking of boxes ( Norcini & Burch 2007). Feedback at its best is an ongoing dialogue between a motivated learner and a supportive and trusted advisor through which goals are identified and strategies for improvement are agreed and reinforced ( Hewson & Little 1998; Urquhart et al. 2014; Eva et al. 2012; Archer 2010; J. M. van de Ridder et al. 2008). Social aspects of this interaction are key to its success and the particular educational culture influences how both learners and tutors approach feedback ( Watling et al. 2014). Medical students have been found to value informal verbal feedback more than formal WBA with written feedback ( Bates et al. 2013; Urquhart et al. 2014). One explanation for this may be that feedback works best soon after the event, especially for a complex task such as consulting with a patient ( Hattie & Timperley 2007; Watling et al. 2008).

Clinical tutors might prefer to dispense with the formal written feedback if it is true that their immediate specific snippets of spoken advice are more likely to be heeded. The destiny of most Workplace Based Assessment (WBA) feedback forms is to be filed in a portfolio of evidence. In their darker moments, tutors sometimes question the likelihood of that painstakingly crafted piece of written feedback ever again crossing the consciousness of the learner ( Higgins et al. 2001).

Conversely, there might be some benefits of written feedback other than ticking the box as evidence for progression. Writing is known to promote information processing in learners ( Quitadamo & Kurtz 2007; Klein 1999). Committing feedback to the written word might improve its quality by a similar cognitive process in the assessor. Medical student and trainee doctor satisfaction with their workplace feedback increases when a written card system is used to prompt feedback ( Ozuah et al. 2007; Richards et al. 2007; Yarris et al. 2011). It is however not clear what lies behind such enhanced satisfaction, and in one study faculty giving the feedback perceived no difference in quantity, quality and timeliness of feedback ( Yarris et al. 2011). The observation of a bias towards leniency in written WBA feedback with a lack of recommendations for improvement warns against equating student satisfaction with enhanced learning ( Prystowsky & DaRosa 2003; Richards et al. 2007; Bandiera & Lendrum 2008) Such findings beg further qualitative research to be understood.

Before we conclude that we are wasting everybody’s time by requiring the completion of feedback forms which have little chance of being read and even less chance of improving performance, let us check for the presence of a baby in the bathwater we are considering throwing out. It is worth taking a closer look at exactly what is happening more widely in WBA systems which involve the generation of written feedback.

## Context

At Keele, we believe that the learning culture should support the giving of useful feedback. We have therefore invested heavily in serial formative WBA of students’ consultation skills including the provision of formal written feedback using a validated assessment and feedback instrument ‘generic consultation skills’ (GeCoS), an iterative quality enhancement process ( Bartlett et al. 2017) and a purpose-designed app and web resource. These resources enable clinical tutors to select the competencies assessed and to add their own free-text comments to relevant student-validated strategies for improvement ( Lefroy et al. 2011; Lefroy et al. 2014). When we found that routine end-of year student evaluation consistently showed greater satisfaction with verbal feedback ( table 1) despite this impressively detailed written feedback they were getting we decided to explore other aspects of this phenomenon.

**Table 1.** Evaluation data student satisfaction Y5 2012-13

How useful was the feedback?	Informal verbal		More formal(verbal and written)	
Location	Primary Care	Secondary Care	Primary Care	Secondary Care
**Students selecting “very useful” and “useful”**	98%	87%	80%	63%

Our research question was: Does the process of completing a written summary of WBA feedback add value for the learner?

We thought that we perhaps had access to data that might address this question in interviews collected for a qualitative study about WBA that we had conducted with our 3 ^rd^ year student cohort in 2012 ( Lefroy et al. 2015).

## Methods

A secondary analysis was made of the transcripts from semi-structured interviews with students who had recently had three WBAs with verbal and written feedback during a four week clinical placement. The data had been collected for a study on the impact of grades in formative WBA ( Lefroy et al. 2015). The methodology of the original study can be seen in that publication. In brief, all year 3 medical students and their GP tutors for the placements of 2012 were invited to participate. Maximal variation sampling was used when selecting interviewees with respect to gender, attainment in the recent OSCE and preference for feedback with and without grades. Semi-structured interviews were conducted at the end of the placement exploring what students felt about questions including their views on WBA. One researcher (JL) conducted the interviews. Interviews were audio-recorded with consent, and transcribed verbatim. The interview schedule can be seen in appendix 1.

Participants in the original study had been asked to consent for their data to be stored for future research. Ethical approval for this secondary analysis was granted by the Keele School of Medicine Ethics Committee.

In the secondary analysis, transcripts from the interviews held in 2012 with 24 Year 3 medical students were examined. The written feedback to 23 of these students was also examined (one student had not received written feedback).

We used a coding process to generate two subsets of data. Two researchers (JL and AH) independently re-read the interview transcripts to identify all references to *verbal/informal/feedback* and to *written/formal/GeCoS feedback* (GeCoS = Generic Consultation Skills - the assessment and feedback instrument used ( Lefroy et al. 2011; Lefroy et al. 2014)). In a grounded secondary analysis, cross-comparison of the data within these two codes was made. Each of the researchers analysed student experiences and views about their feedback discussions with tutors and their written feedback summaries looking for direct comparisons made by students or interpreting the differences in their talk about each. The second dataset was the written feedback provided in the WBAs of consenting tutors. This was also examined to compare and contrast it with the students’ descriptions of their informal verbal feedback. When the student recalled their feedback we checked to see whether the same terms were used in the written feedback. The free text comments in the written feedback were also checked for similarity with the phrases embedded in the GeCoS instrument.

Researchers were already familiar with the data following the previous study and were thus immersed within it. The recoding and the new lens of comparative analysis were used to make the familiar unfamiliar again. Following their independent analyses the researchers then met to discuss their findings. Any disparities in their findings were discussed to reach consensus.

## Results

Four main themes relating to the research question were identified: the value of verbal feedback, written feedback being felt to be relatively superfluous, the augmentation effect on verbal feedback of having a written summary to produce, and the adoption of the language of the formal instrument in both verbal feedback and free text written feedback. These themes are expanded below.

### Verbal feedback was valued

Verbal feedback on their consultations were seen by students as supportive, rich in content, immediate, specific and desirable.

F10: *My doctor was very good at (feedback) if he was sitting in on a consultation as soon as the patient had gone out we went through what was good, what was bad, what could be improved sort of thing and I think having that immediate feedback on it while it’s fresh in your mind still erm I think that was really good. I think yes definitely the immediate feedback really helped with me cus then the next person who came in two minutes later, I was thinking about what he’d just said and I was bringing that into the next consultation straightaway.*

The feedback dialogue also gave more opportunity for the student to probe the tutor about what they meant by their feedback. For example this student was initially unhappy with his critical feedback but came to appreciate it after some discussion:

M1 *he seemed to notice at times that I was quite sort of annoyed maybe or a bit put down by the criticism I got and .. he said things like oh .. I hope you don’t think I’m being too critical of you or whatever. And we had a chat about it so I don’t know if that influenced him to be, to give a bit more praise.. cus I was saying that he was comparing my consultation skills to sort of his very high standards that he has. Erm and he said oh who do you want to be compared to? And I said well I suppose, I think about comparing myself to the other members of my peer group and he just said, you shouldn’t compare yourself to people in your group. You should be comparing yourself against like the highest standards you can be compared to.*

The friendliness of the verbal feedback was appreciated:

M7: *I felt he was being more almost as if he is telling from experience, as a friend - one person to another; almost like that information you give on the side kind of thing (laughing) so he was telling you the crucial details. He was like “really do this cus this is important”*

### Written feedback was less valued

Most felt that the written feedback was superfluous except as a record of what had been spoken, and this record was not always welcomed.

Interviewer: *And how do you view workplace-based assessment? So those GeCoS assessments that you had?*

F23: *I thought they were a bit silly. (laughs) I just found it a bit odd. Because the doctor’s given me feedback on my consultation skills anyway, I didn’t feel I needed the GeCoS on top of it. It just put added stress into a situation which really didn’t need any more stress.*

This student went on to explain her preference for informal feedback:

F23: *It was informal, there wasn’t anything to fill in or any grades put on it or any.. points to improve. It was just sort of said “Oh here’s an idea, you should just look at this or go away and read this” rather than “this is what you have to do”..*

And of the stress:

F23: *just knowing that someone is .. assessing you. And that it’s going to be written down somewhere on record, I think makes it more stressful than just.. being an informal thing that.. only really you and the person in the room knows.*

They also felt that the written feedback was constrained by the assessment and feedback instrument used for the formal assessment.

F13: *I think it was mainly cus I had just presented these cases and the questions weren’t.. really relevant to the history that I’d just presented.. I think we both found it quite difficult to tick boxes.*

The inability to get an immediate query about written feedback answered was a problem for a few students:

M1: *I thought oh what am I doing wrong. I must be doing quite a lot wrong. Erm so yes, I didn’t really know what to think after that cus it was more, it was quite rushed going through it and.. it wasn’t discussed with him at the time. I received it via e-mail*

Several students had not engaged with their written feedback:

F13: *I can’t remember off the top of my head but I did get given a form, summary form thing I think somewhere.*

There were exceptions to the opinion that verbal feedback was better than written, especially from students who wanted specific, well-thought-out feedback or those who wanted grades:

M22: *There was a lot more focus on what you can do next time, in a very clean bullet -pointed note. It wasn’t a sort of wishy washy you know “Yeah you did OK but you should..” It was like “Do this next time” or “don’t do that”.. It’s very constructive to know exactly what to do differently next time.*

M6 *I mean with feedback it’s more of a like subjective thing so you’ll just, you’ll just hear you know a few words, you might forget them in a few weeks but with the grades like it’s written down.*

### Having to produce a written summary has the effect of augmenting feedback

Students reported that their clinical supervisors undertook multiple informal assessments with verbal feedback before completing each formal written assessment. There was often a three-stage feedback process of immediate post-consultation feedback, summary discussion and written advice. While they preferred verbal feedback to written, they acknowledged that their assessors took great trouble over the formal assessment and often discussed it with them at length. This additional discussion and the written feedback felt qualitatively different from the immediate feedback - more constructive.

M12: *I got feedback straight after. Sort of just face to face, him telling me about the consultation..*

*Then a bit more specific feedback - more detailed - particularly after the assessment. Just about history taking skills and questions I should ask or ways I could phrase things.*

*And then I got more sort of written feedback quite soon after. It was always quite prompt.*

And:

F21 *Before we went on the computer she sort of said what did you do well? What might you want to change in the future? And then she sort of gave me a brief thing as well and then we went on the computer and looked at the more specific answers we could have - the strategies.*

And:

M18 *I think the main thing was that she talked me through it, as opposed to me just reading it. So even though it was pretty thorough what she said, and it was really good on GeCoS but the fact that she talked me through was really helpful, and allowed me to - sort of - cos sometimes when you read something you have your own perceptions and you think that: Maybe she’s saying this; Maybe she’s saying that. But it sort of clarified to me what she actually meant and what she wanted me to say.*

Even this student who preferred the informal feedback could see that his tutors were adding value to it when they wrote it down:

M12 *I think perhaps because we’d always discussed it face to face in some form before getting the written feedback, you sort of could appreciate what was written there more because you’ve already discussed it.*

*Personally I prefer face to face feedback. But I like that you’ve got a written record so you remember what’s come back. You might not remember everything that’s said to you when you have face to face feedback.*

*And I think when they’re writing feedback more formal and written, they’ve had a bit more time to think about it. And any sort of constructive elements to it were always more in the written.*

*So when we were discussing face to face it was more sort of just noticing what had gone on and just talking a little bit about it. But when it was written it was always how to take that forward a bit more.*

### The language of the formal instrument was being adopted in informal verbal and free text feedback

What was notable in students’ descriptions of their informal feedback was that it did correlate closely to their written feedback. This may indicate that the assessors were adapting to using the same terms in informal feedback discussions which they had been required to use in the formal assessment instrument. This is certainly the assumption made by student F11, and she approved, considering the feedback as more valuable because it aligned with their curriculum:

F11: *I think it was really good in the GP’s because obviously they were kind of told what to do and they were told, they were given the guidelines on how to do it. So it was quite helpful.. It was nice having someone watching me do it so that I could, you know, get good feedback.*

We were able to corroborate this as illustrated by these three examples from comparative analysis of the student interviews with their written WBA feedback:

Student F21 described the informal verbal feedback received:

F21: *After each patient left he told me what he thought of the history - the good bits and bits missed - and my examination.*

Interviewer: *What feedback do you recall?*

F21: *I asked multiple questions of the patient.*

In her written feedback summary the tutor selected the same concept of avoiding multiple questioning from the pre-formulated strategies for improvement (see figure 1):

**Fig 1.** Extract from written feedback to student F21:

Opportunity for improvement:HISTORY PROCESS: Skilled use of questioning including open and closed questions	
GeCoS Strategy selected:	Don’t use ‘double’ or ‘nested’ questions e.g. ‘What is your pain like and how long have you had it?’ ‘Is your appetite normal and have you lost weight?’

Student F24’s tutor used the language of the assessment instrument in free-text feedback reminding her of the previous week’s selection from the GeCoS strategies for improvement in her feedback (See figure 2 & 3).

**Fig 2.** Week 3 WBA extract for student F24:

Opportunities for improvement:OPENING: Establishes agendas	
GeCoS Strategies selected:	Identify the patient’s agenda. Develop a range of opening questions for different situations with which you are comfortable
	Check that your understanding of the patient’s agenda is complete: ‘Is there anything else you would like me to do today’

**Fig 3.** Free text comment in WBA for student F24 week 4:

*“(You) reflected on last week’s feedback and started to clearly establish patient’s agenda at start of each consultation”*

In free text feedback to student M5 his tutor used the language of the assessment instrument to reinforce the relevance of the selected strategy to the student’s need to improve ( figure 4):

**Fig 4.** Week 3 WBA extract for student M5 and relevant GeCoS strategy:

Opportunities for improvement: HISTORY: History content - Details of symptoms Free text comment: *“You missed a few key elements to the history such as weight loss or gain. Using SOCRATES would have helped”*	
GeCoS Strategy selected:	Use a mental checklist such as SOCRATES (which is useful for many symptoms) to clarify the presenting complaint(s)

## Discussion

The secondary analysis of this data set has confirmed the high value placed on verbal feedback from their GP tutors by our students. This mirrors previous research in which verbal feedback was viewed more positively than written ( Urquhart et al. 2014; Bates et al. 2013).

This preference for verbal over written feedback appears to relate to its immediacy and also to feedback as a social interaction,( Hattie & Timperley 2007; Watling et al. 2008) which both work better face to face than in writing. For example, verbal feedback enables reciprocity of dialogue in which students can act quickly to resolve questions and conflict. By comparison one-way written feedback seems less satisfactory. Another socially important aspect described by some students is the intimacy of verbal feedback: it appears to be less threatening than the written (and potentially public) “assessment” even in this entirely formative situation. These data, however, suggest a paradox: written feedback may contain more useful advice than verbal feedback (M12 above) yet is still not preferred. Understanding feedback as a social interaction and the value students place on the interaction may help us to resolve this paradox. We have demonstrated further social attributes of verbal feedback which make it preferable to written feedback such as keeping criticism private and allowing dialogue about it. In summary, verbal feedback was valued more highly than written because of its richness, immediacy, intimacy and interactional effects which written feedback cannot match.

Notwithstanding this very clear preference for verbal feedback, these data suggest that the requirement to provide ‘formal’ written feedback may be augmenting ‘informal’ verbal feedback. The description given by students of the WBA process suggests three stages - immediate feedback, then a discussion in which feedback is summarised and strategies suggested, and finally receipt of the written feedback. The feedback discussion was linked by students to the requirement for formal assessment and although this qualitative data cannot prove that students in a similar clinical placement without the requirement for written feedback would get less feedback in total, the large quantity of feedback described here contrasts with the usual student and graduate complaint of insufficient feedback ( J. M. M. van de Ridder et al. 2008; Reddy et al. 2015). In this study, the verbal feedback also appears to be aligned with the written feedback which in turn is aligned with the formal consultation skills curriculum ( Lefroy et al. 2011; Lefroy et al. 2014) which we consider to increase its educational value. We consider that production of the formal written WBA using the online instrument to facilitate corrective feedback enabled tutors to give feedback aligned with the curriculum and in the language of medical education. It may also help to familiarise tutors in the language of consultation skills assessment which they then use in their feedback. Tutor engagement in feedback is key to its success ( Watling et al. 2008). Requiring formal WBA will have ensured tutor engagement. Giving tutors the feedback tools and language may have encouraged this wealth of feedback. Without this realisation, the medical education intervention of mandatory written WBA might be seen as of limited value. We acknowledge that these two findings are tentative but, if they are confirmed, they extend knowledge about complexity of the feedback and the culture and environment in which it is embedded.

Elements of a learning culture which support the exchange of meaningful feedback have been identified as: a systems approach; supporting the development of trusting supervisor-trainee relationships; using video review with feedback; promoting communities of practice in which feedback is routine, regular and valued; making sure that the trainer’s role and the learners’ educational objectives are understood, and ensuring that competency in providing feedback is maintained and improved by reflection and refresher courses ( Lefroy et al. 2015). The system of regular formal formative WBAs with training and a feedback instrument which we have introduced appears to have made an important contribution to the development of a such a learning culture across our network of 100 teaching practices( Bartlett et al. 2015). Sometimes you have to wait to see and then look for the benefits of an educational intervention.

### Strengths and weaknesses

This is a secondary analysis of data. We consider in this case this is a strength. Data was collected to explore the impact on students of having grades with their written feedback. The interview schedule asked about recall of informal feedback to understand whether there was any difference in the discussions with tutors when grading was part of the process. This provided a good source of data for this subsequent research question.

The main weaknesses in this study are first that it is located firmly within one school which has invested heavily in its workplace assessment and feedback culture and second because it is a secondary analysis, we have not been able to examine causality. There were no parallel tutor interviews in that study to corroborate our inferences about staff development impacts of the WBA system and app. Whether or not these virtuous impacts would be driven by simply requiring the production of written feedback without a considerable parallel development of tutors is unknown ( Bartlett et al. 2015).

## Conclusions

Although the WBA was entirely formative for these students, they wanted to perform well and they reported that their tutors were engaged with helping them. Students recalled expansive and rich feedback and this was mirrored in their written feedback. The feedback process may have been enhanced by the requirement for written WBA. Furthermore, assessors have used the school’s language for assessing and giving feedback on consultation skills. Using the school’s app for producing written feedback summaries may have had a staff development effect of internalising the medical education language. Consequently students also had verbal feedback which was closely aligned to the formal curriculum. This at least in part explains the disparity in student satisfaction with verbal and written feedback. However, we consider that this is likely to be a positive unintended consequence of the requirement to complete three formal workplace-based assessments. Justification for medical education interventions needs to look beyond the obvious or immediate. Here is one example of a possible secondary beneficial effect of an intervention.

Feedback has a culture ( Watling et al. 2014), a culture that it seems is open to influence in unexpected ways. The introduction of this system supporting mandatory serial formative WBAs seems to have changed the feedback culture in a beneficial way for our students.

We conclude that even if students never read their written feedback, the process of generating it is worthwhile and should not be abandoned. The practice of reflecting on the feedback (using the written summary as an aide-memoire) is likely to add to the formative outcomes but this remains to be proven.

## Take Home Messages

What we found:

• Tutors appeared to be engaged based on student report
• Language in the verbal and written feedback was aligned with the curriculum
• Students recalled expansive and rich feedback and this was mirrored in the written feedback
• The feedback process may have been enhanced by the requirement for written WBA
• The curriculum-aligned language may have been promoted by use of a feedback app

## Notes On Contributors

**Janet Lefroy** is a Senior Lecturer in Medical Education at Keele University. She is a UK general practitioner and leads consultation skills teaching and learning at Keele’s School of Medicine. She has carried out action research developing assessment and feedback for consultation skills.

**Ashley Hawarden** is a Clinical Teaching Fellow at Keele University. At the time of the study he was an undergraduate medical student. His research interests include assessment and feedback for clinical and consultation skills.

**Simon P Gay** is Director of Education Governance and Clinical Associate Professor in Medical Education at the University of Nottingham Medical School and Undergraduate Curriculum Advisor at Keele University School of Medicine. He is a UK general practitioner whose research interests include clinical reasoning and consultation skills development.

**Robert K McKinley** is Professor of Education in General Practice at Keele University and a practicing general practitioner in the UK. His major research interests are workplace assessment and education in primary care.

### Contributorship

RKM and JL had the original idea for this study. JL and AH conducted the analysis. JL wrote the first draft of the paper and AH, SPG and RKM all contributed to subsequent drafts. All have approved the final version. JL and AH are guarantors for the paper.

## Appendices

**Interview guide for the study “Grades in formative workplace-based assessment - a study of what works for whom and why”:**

This interview is about your consultation skills. How did you get on in the Consolidation of Clinical Skills block?

How good are your consultation skills? How are your consultation skills progressing? What is your self-reference? (What do you compare yourself to?)

What has affected your progress? (Helped? Hindered?)

How do you view Workplace-Based Assessment?

What happened in each of your 3 WBAs? (What feedback do you recall?)

What did you do with the WBA feedback? (What effect did the WBA feedback have on you?)

(Where did you focus your attention after the feedback?)

Were there actions you could have taken arising out of the feedback you were given?

Why did you choose all grades/grades for your week 4 WBA/no grades for your week 4 WBA?

How important was it to you?

Was there any difference in how you responded to feedback with and without grades? Why?

Is it helpful or unhelpful to be graded by the standard you should be on graduating from medical school?

Did the feedback resonate with you? Fair? Unfair? Too kind?

(What were the differences between your self-assessment and the grades, verbal and written feedback you got?)

How did the feedback and grades compare with previous assessments you have had?

Would it help to be allowed to choose whether to have grades or not with your future workplace-based assessments?

What difference would it make?

Would you want grades? Would they be good for you? What are the advantages and disadvantages?

Did you discuss the choice with your tutor? (What effect did that have?)
