# Supplementary material for: Does formal workplace based assessment add value to informal feedback?
Source: MedEdPublish (2016). 2017 Feb 9;6:27. [Version 1] doi: 10.15694/mep.2017.000027 (PMC10885233; doi:10.15694/mep.2017.000027)
Supplement: Peer review history

# Reviewer report: Reviewer response for version 1

**DOI:** 10.21956/mep.19439.r28608

**Published:** 2017-02-10

**Authors:** Richard Hays

**Affiliations:** 1 James Cook University

This review has been migrated. The reviewer awarded 5 stars out of 5 This paper reports very clearly some interesting information on feedback, which is a hot topic in medical education. The key point for me - perhaps we could call it the 'feedback paradox' - is that learners may prefer rapid, verbal feedback in a discussion, even though written feedback may ultimately be more helpful in terms of raising more issues. Was there any evidence for a difference here, depending on whether the feedback was positive or negative? Happily, most places use both verbal and written feedback strategies and there is increasing pressure for us to document formally such information. The feedback app sounds like a step forward in gathering more information, so long as it does not focus too much on ticking boxes with pre-defined statements.

Reviewer Expertise:

NA

No decision status is available

# Reviewer report: Reviewer response for version 1

**DOI:** 10.21956/mep.19439.r28605

**Published:** 2017-02-10

**Authors:** Subha Ramani

**Affiliations:** 1 Harvard Medical School, Brigham and Women's Hospital

This review has been migrated. The reviewer awarded 4 stars out of 5 I enjoyed reading this paper and concur with other reviewers' comments. It is an important topic, through decades of staff development on feedback- feedback quality is still rated as sub-optimal. Written comments on assessments are another aspect of the feedback exchange and these seem to be less valued by learners for very good reasons. Written comments are best used as a documentation of what occurred in person so learners can review later, however, the comments should not come as a surprise or shock. They should have had this conversation already. Also, goal directed and action oriented feedback is essential given David Boud's definition that feedback is feedback only when it results in behavior change.Couple of comments:- The way the paper is written, readers could interpret that feedback and assessment are one and the same. They are quite distinct. They may occur in the setting of assessment, but the process and purpose are different. I agree that verbal feedback is necessary to close the loop of WBA, but it is separate.- The authors should have referred to work by Ginsburg et al, a rich set of research papers addressing the non-specificity of written narratives on assessment of learners.In summary, this work will be useful to all medical educators because assessment and feedback are important to a wide spectrum of teachers from tutors, clinical supervisors to educational course leaders. Additional work in this area would be illuminating.

Reviewer Expertise:

NA

No decision status is available

# Reviewer report: Reviewer response for version 1

**DOI:** 10.21956/mep.19439.r28606

**Published:** 2017-02-09

**Authors:** Trevor Gibbs

**Affiliations:** 1 AMEE

This review has been migrated. The reviewer awarded 4 stars out of 5 A very interesting paper that covers an important area of assessment- feedback, probably an area that still requires improvement. I was not surprised by the students preference of verbal over written feedback, and would expect the faculty to response similarly, but maybe for other reasons related to workload and time allocated for feedback. There are many features that enhance feedback, but I feel that combining verbal feedback with written evidence of what was said and, as my co-reviewer point out, the important feed-forward element makes feedback more acceptable and greater utility. There is also the legal requirement of charting what was discussed and agreed upon, which supports or refutes any arguments if students are either borderline or failures.A very useful paper on the subject and one which should be read by those involved in the process.

Reviewer Expertise:

NA

No decision status is available

# Reviewer report: Reviewer response for version 1

**DOI:** 10.21956/mep.19439.r28607

**Published:** 2017-02-09

**Authors:** Deborah Murdoch Eaton

**Affiliations:** 1 University of Sheffield

This review has been migrated. The reviewer awarded 5 stars out of 5 Very interesting paper. There are often differing perceptions of learners as to preferences for written or verbal feedback, and many identify verbal feedback in the clinical environment is preferred because of the immediacy in relation to their clinical practice. This study is important becuase of the inference that the written feedback prepared the feedback giver in the quality of the verbal feedback,, and facilitates the feedback conversation. it also identifies good practice ie that written record provided may be valuable to learners to refer to later,. A further valuable study might be to evaluate whether "feed forward" ie utilisation of the written feedback was enhanced by the addition of supplementary verbal feedback and encourages learners to refer back to the written feedback.

Reviewer Expertise:

NA

No decision status is available

# Reviewer report: Reviewer response for version 1

**DOI:** 10.21956/mep.19439.r28604

**Published:** 2017-02-09

**Authors:** John Dent

**Affiliations:** 1 AMEE

This review has been migrated. The reviewer awarded 4 stars out of 5 I liked this well-presented report.The topic is interesting and the comparisons between formal written feedback and informal verbal feedback are clearly made. Verbal feedback is popular because of its immediacy, its opportunities for discussion and because of its personal touch. The disadvantages accorded to written feedback were it being superfluous and not always closely linked to the situation under review.Interestingly, from the reviewer's perspective,the exercise of having made a written review first informed their subsequent conversation in the verbal feedback session. Having some clarity of thought before talking seems to be a good idea!

Reviewer Expertise:

NA

No decision status is available
